# An ensemble Kalman filter with rescaling disaggregation for assimilating terrestrial water storage into hydrological models

**DOI:** 10.1038/s41598-025-13602-2

**Published:** 2025-08-06

**Authors:** Leire Retegui-Schiettekatte, Maike Schumacher, Fan Yang, Henrik Madsen, Ehsan Forootan

**Affiliations:** 1 Geodesy Group, Department of Sustainability and Planning, Aalborg University, Rendsburggade 14, 9000 Aalborg, Denmark; 2 DHI A/S, Agern Allé 5, 2970 Hørsholm, Denmark

**Keywords:** Hydrology, Hydrology, Hydrogeology

## Abstract

Assimilating satellite-based Terrestrial Water Storage (TWS) observations can improve the vertical summation of water storage states in hydrological models. However, it can degrade individual storage compartments or hydrological fluxes, limiting the applicability of TWS Data Assimilation (DA) for water management and flood monitoring. This issue arises from the ensemble-based TWS update disaggregation approach used by DA techniques like the Ensemble Kalman Filter (EnKF). Thus, this study makes two key contributions. First, we introduce a novel analysis method that provides quantitative and qualitative insights into how individual storage compartments are affected during TWS DA, by examining the sign and magnitude of the individual storage updates and their responses. Second, we propose a new disaggregation approach, EnKF-R, which “rescales” the individual storage of model compartments to match the updated TWS, avoiding the use of ensemble statistics within the disaggregation process. The EnKF-R approach was tested in two climatologically different river basins and validated against both synthetic and real independent data. Our results show that EnKF-R produces similar TWS estimates to the classical EnKF while reducing degradations in individual water storage compartments and with lower computational cost, making it a promising alternative. Limitations regarding spatial continuity and uncertainty estimation require further developments.

## Introduction

Large-scale hydrological models are tools to simulate the water cycle, and they provide essential information for various applications. However, simulation skills of the available models (from the simplest to the most complicated) are limited, especially to represent water storage and changes in water flow, at various time scales^1,2^. This can be due to errors in forcing (input) data and uncertain parameters, as well as to a simplified representation or misrepresentation of complex hydrological processes within the model structure^3^. To improve the closeness of these simulations to reality, data-model fusion techniques such as sequential Data Assimilation (DA) are found to be efficient^4^.

Satellite-based hydrological observations are especially suited for large-scale hydrological DA due to their broad coverage and open availability, and have the potential to contribute to more precise hydrological simulations^5,6^. Since 2002, changes in Terrestrial Water Storage (TWS, a vertical summation of all components of land water including ice, snow, vegetation water, wetlands, lakes, rivers, soil moisture and groundwater) have been measured by the satellite gravity mission GRACE (Gravity Recovery and Climate Experiment)^7^ and its Follow-On mission (GRACE-FO)^8^. The official GRACE and GRACE-FO products are provided as monthly solutions with a spatial resolution of around 300 km with global coverage. Changes in TWS represent the net effect of climate variability and change, as well as anthropogenic impacts on the water cycle^9–11^. The sensitivity of GRACE(-FO) TWS to groundwater changes also makes it very attractive for assessing deep-water resources and improving model simulations^12–16^.

**Fig. 1:**
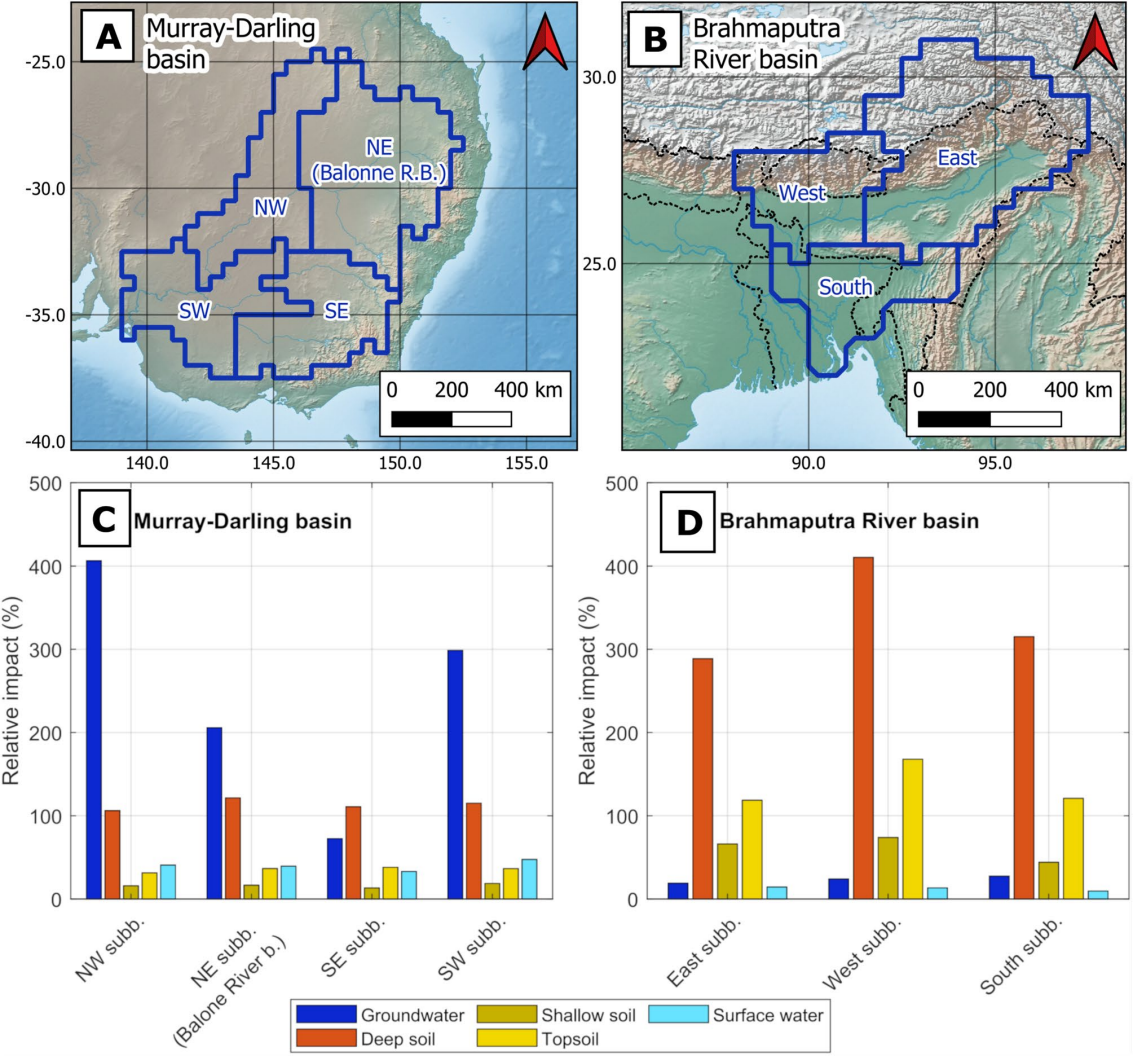
(**A**, **B**) Map of the Murray–Darling basin (**A**) and the Brahmaputra River basin (**B**). The sub-basins in which TWS observations are averaged and assimilated are outlined in dark blue. Rivers are represented in light blue and political boundaries are outlined in black dashed line. (**C**, **D**) Relative impact of TWS DA in individual water storage estimates, expressed as a percentage of the variability of the signal for each compartment (see Supplementary Tables S1 and S2). The relative impact is computed based on the results obtained through the classical EnKF approach, in the Murray–Darling basin (**C**) and Brahmaputra River basin (**D**). (**A**) and (**B**) were created using QGIS 3.40.0 (qgis.org). The background maps were downloaded from Natural Earth public domain dataset (www.naturalearthdata.com). (**C**) and (**D**) were generated using Matlab 2020b (se.mathworks.com/products/matlab.html). The figures were put together using GIMP 3.0.2 (www.gimp.org).

TWS observations are typically assimilated as monthly spatially averaged fields (averaged on a sub-basin scale^15,17,18^ or grid scale^19–21^). The integration of TWS into hydrological models through sequential DA is performed by initializing and running the model forward to reach a timestep with an available observation. The model state update is then performed by computing a weighted average of the observation and the model prediction of TWS, with weights depending on the uncertainty of both the model and observations. The computed update is then distributed among the model state variables. Finally, the updated model state is used to re-initialize the model, which runs until the next observation becomes available^22^. A mathematical description of the DA process can be found in “Data assimilation” section of this manuscript.

The Ensemble Kalman Filter (EnKF) approach^23^ is one of the most popular sequential DA strategies because it is broadly applicable and its implementation in the form of matrix operations is relatively straightforward. Previous studies have shown that the EnKF-based DA approach for integrating monthly TWS into models can introduce long-term trends and adjust the seasonal variability of their storage simulations^15,17,20,21,24,25^. The DA-based TWS has the realism of GRACE(-FO) observations and preserves the finer spatial and vertical resolution of the models^17^. Thus, DA can be useful for various applications^26^, such as groundwater forecasting^27^, detecting the irrigation footprint^28^, and predicting and monitoring flood events^18,29^. However, for a general applicability of the outputs, the impact of TWS DA on individual water storage and flux estimates is very important. For example, for river flow monitoring applications, updates of soil moisture, surface water, and river discharge must be critically evaluated^30–32^. For water resource management, updates of soil water and groundwater storage are key variables to control^15,16,33^. Several studies report that the TWS DA (including the basin-averaged and gridded schemes) is likely to cause degradations in individual model estimates such as soil moisture^25,34^, river discharge^32^, and evapotranspiration^33^.

Degradations in individual model components are caused by an inadequate distribution of TWS DA updates among model variables (that is, different water storage compartments and grid cells). This separation poses an inverse problem that is under-determined, meaning that it has multiple valid solutions, as different vertical and horizontal water storage distributions can lead to the same spatially averaged TWS value. In the EnKF approach, this problem is solved by setting additional constraints based on ensemble statistics, which represent the correlations between the model state variables and the observed TWS. This means that model state variables with strong correlations with TWS receive a large update with the same sign of the TWS update, while strongly anticorrelated ones also receive a large update but with an opposite sign. In addition, variables with high uncertainties receive a larger update than those with lower uncertainties. A mathematical description of this process is provided in “A two-step decomposition of the EnKF” section. Although this approach should work well for large ensemble sizes, the high computational demand of hydrological model runs restricts the ensemble size to typically 20-30 members^15,17,20,25^, preventing a robust computation of correlations^35^. Therefore, possible spurious correlations in the ensemble statistics can lead to an incorrect disaggregation of TWS updates into individual storage components. Additionally, with such small ensembles, the ensemble generation approach can strongly condition the correlations and hence the performance of TWS update separation.

To mitigate the impact of spurious correlations in TWS DA, a typical approach is covariance localization, which damps model correlations of distant grid cells given a predefined radius^35,36^. Previous studies suggest that such an approach might help reduce the degradation of individual water storage compartments^18^. However, finding an appropriate radius is not straightforward, as a too large or small radii can negatively affect the results^18,37,38^. Additionally, for the assimilation of non-local observations such as the spatially averaged TWS, localization needs to be implemented in the model space^39^. This operation involves $N_{var}^2$ multiplications, where $N_{var}\gtrsim 10000$ is the number of model variables, leading to a substantial increase in the computational load. Furthermore, localization may not completely eliminate the undesirable effects^18^.

The objective of this study is twofold: (i) to develop a method to analyze the impact of the TWS DA updates on individual water storage compartments and (ii) to propose and evaluate an alternative filter that eliminates the dependence on ensemble statistics during the disaggregation of the TWS update. To address the first objective, we introduce a novel evaluation approach that focuses on quantifying the update and response of individual water storage estimates to the TWS DA. Regarding the second objective, we develop a new filter that maintains the sequential EnKF framework while implementing a revised TWS disaggregation method, which is known here as EnKF Rescaling (EnKF-R). This new approach solves the inverse problem by adding a storage-based constraint, where the update of each individual water storage component should be proportional to the amount of water stored there. The EnKF-R approach is based on the intuitive idea that compartments that have a higher amount of water storage might also be the ones contributing the most to TWS dynamics and, therefore, should receive a larger portion of the DA update. We acknowledge that this assumption might not be satisfied in all basins and periods and, therefore, in this study we aim to test the adequacy, applicability and performance of this new approach compared to the classical ensemble-based approach.

The manuscript is organized as follows. In “Proof of concept in the Balonne River basin” section we present a proof of concept of our approach by studying the impact of the TWS DA on groundwater estimates in the Balonne River basin (Australia). In “Comparison and validation of EnKF and EnKF-R” section, we extend the presented analysis to two basins with very different hydrological regimes: the Murray–Darling basin (Australia) and the Brahmaputra River basin (South Asia). We consider the impact of the TWS DA on different water storage compartments and validate the results through synthetic experiments, as well as against independent data. The main results, as well as the benefits and limitations of EnKF-R are discussed in “Discussion” section. Finally, a detailed description of the data and methods used in this study is available in “Method, model and data” section.

## Proof of concept in the Balonne River basin

We first present a proof of concept for the Balonne River basin (North–East of the Murray–Darling basin, Australia, Fig. 1A). This section aims to introduce our novel approach and to provide guidelines for interpreting the observed results.

**Fig. 2:**
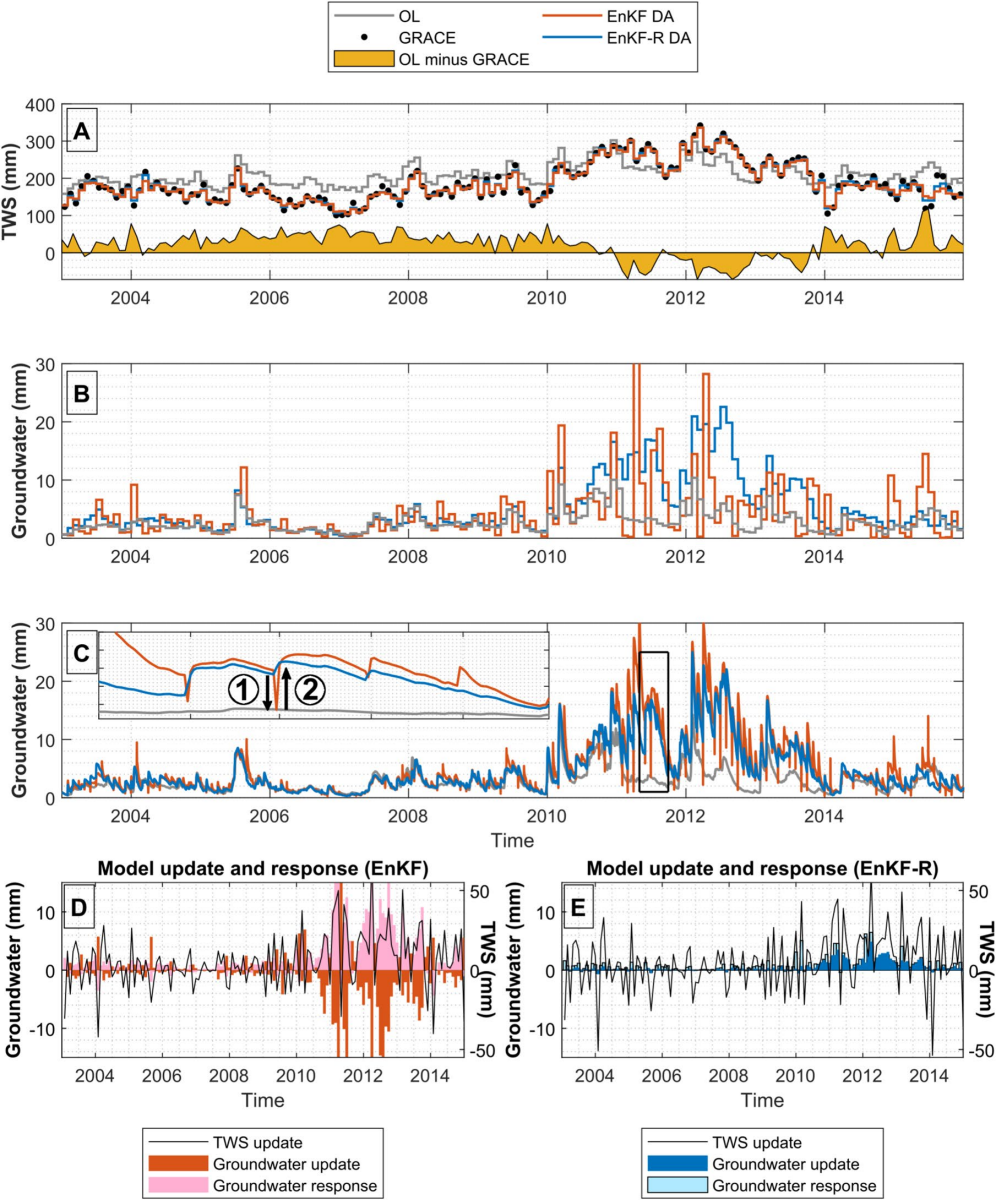
Results of TWS DA experiments in the Balonne River basin. (**A**) The monthly W3RA (OL, Open Loop) and GRACE TWS signal and their difference prior to DA, as well as TWS estimates issued from EnKF and EnKF-R DA approach (mostly overlapping). (**B**) Monthly groundwater estimates obtained by EnKF and EnKF-R DA. (**C**) Daily groundwater estimates for both experiments. The upper left box in this plot shows the detail for the period May–September 2011, with arrows indicating the model update (1) and model response (2). (**D**, **E**) Monthly groundwater update and response dynamics for EnKF and EnKF-R, respectively. The TWS update dynamics are also shown as a reference. Figures generated using Matlab 2020b and modified using GIMP 3.0.2.

During the period 2003–2015, GRACE observations show that the TWS signal in this basin is mainly governed by inter-annual variability, with a dry period corresponding to the Millennium Drought (2001–2009) that affected the entire Murray–Darling basin, and a pronounced posterior recharge of 88 mm of TWS due to wet conditions in 2010 and 2011^40^ (Fig. 2A). The original estimates of the W3RA model are found to underestimate the severity of the Millennium Drought by 37 mm on average, and underestimate the recharge by − 2.5 mm. We therefore assimilate monthly TWS through a classical EnKF approach to improve the representation of inter-annual variability (more details on the method are available in “Implementation of the DA experiments” section). After DA, the inter-annual variability is significantly modified in relation to the GRACE observations, reducing the mean difference during the dry period to 1.4 mm, and that of the recharge period to 1.7 mm (Fig. 2A). These results reflect a good fit of the model to the observations and are similar to those obtained in previous experiments with the classical EnKF in the same area^15^.

We will now have a look at the “update” of these compartments (i.e. how they are affected by TWS DA) and their “response” (i.e., how these compartments react to the update introduced by the DA process); see “Evaluation: measuring model update-response dynamics” section for their mathematical formulation. We first quantify the relative update of TWS DA in each water storage compartment by dividing the Root Mean Squared (RMS) magnitude of the updates by the standard deviation of the time series for each compartment (Fig. 1C). This relative update indicates the extent to which the overall dynamics of the storage compartment is modified by the TWS DA process. For this proof of concept, we will focus on the compartment that gets the highest relative update, that is, the groundwater (200%), while other compartments are addressed in “Comparison and validation of EnKF and EnKF-R” section. The most significant impact of DA occurs during the recharge period (Fig. 2B, red line), with higher groundwater levels (on average 3.7 mm) compared to the original model estimates (gray line). This aligns with the positive shift in TWS from 2010 to 2011. However, DA also causes sharp groundwater variability during this period, with large 30 mm recharge and losses between subsequent months.

These updates seem physically inconsistent with the general water storage recharge context, which the model should reflect due to high rainfall input, and suggest inadequate TWS disaggregation during DA. To understand how the model accommodates sharp DA updates, daily time series are shown in Fig. 2C. The monthly groundwater update is applied on the last day of each month (see “Implementation of the DA experiments” section). A plot of May–September 2011 indicates that the classical EnKF introduces sharp negative updates (arrow 1) at the end of each month, which the model immediately counteracts in the first timestep after receiving the update (arrow 2) to return to its natural dynamic.

The monthly groundwater update (arrow 1) and response (arrow 2) time series were calculated for the entire time span (see “Evaluation: measuring model update-response dynamics” section) and are shown in Fig. 2D. The TWS update (black line) is included as a reference. The time series exhibit a regular pattern in terms of sign. The groundwater update (dark red) has the RMS magnitude of 4.6 mm and generally has an opposite sign to the TWS update, indicating that the groundwater storage compartment is anti-correlated with TWS in the ensemble representation of the current DA implementation. In contrast, the groundwater response (pink) has a sign opposite to and magnitude similar to the update (4.9 mm), suggesting that the model response typically counteracts the DA update. This response is particularly strong during 2010-2011 and indicates a potential incoherence of the updates with the physics represented within the model.

To mitigate this problem, we introduce the novel DA approach, EnKF-R, which retains the main features of EnKF but differs in the TWS update distribution. Instead of using ensemble statistics, EnKF-R distributes the TWS update increment in proportion to the amount of water stored in each compartment, effectively rescaling them (see details in “Novel rescaling disaggregation scheme” section). The DA experiment is repeated using the new method, and the results show that both EnKF and EnKF-R lead to almost identical TWS estimates (RMS Difference, RMSD, of 4 mm, see Fig. 2A, blue line). However, during the recharge period, EnKF-R avoids sharp spikes in the groundwater (Fig. 2B), resulting in smoother groundwater compartment dynamics compared to EnKF. Daily time series confirm the absence of sharp spikes with the novel approach (Fig. 2C).

The groundwater update and response time series for the EnKF-R are shown in Fig. 2E. The magnitude of the updates is much smaller than that of EnKF (RMS magnitude of 1.0 mm), and the sign is overall the same as that of the TWS update (see more details in Supplementary Section S1). The magnitude of the model response is also significantly lower with EnKF-R (RMS magnitude of 1.4 mm) and displays a sign similar to the update. It should be mentioned that a totally neutral model response should lack DA-related patterns in the sign, and therefore the sign of EnKF-R groundwater response might indicate some conflicts with model physics. A possible explanation could be that the EnKF-R introduces excessive updates in the deep soil compartment, later transferred to groundwater through percolation processes. However, the deep soil water dynamics is found to be similar for both EnKF-R and EnKF (“Experiment 1: Murray–Darling basin” section). Therefore, EnKF estimates are probably also affected by a similar problem, but this effect is masked by the larger response generated by groundwater updates. In summary, EnKF-R is found to reach TWS results similar to EnKF while triggering smaller groundwater responses, and therefore might be more respectful towards the physics represented within the model than EnKF.

## Comparison and validation of EnKF and EnKF-R

### Experiment 1: Murray–Darling basin

**Fig. 3:**
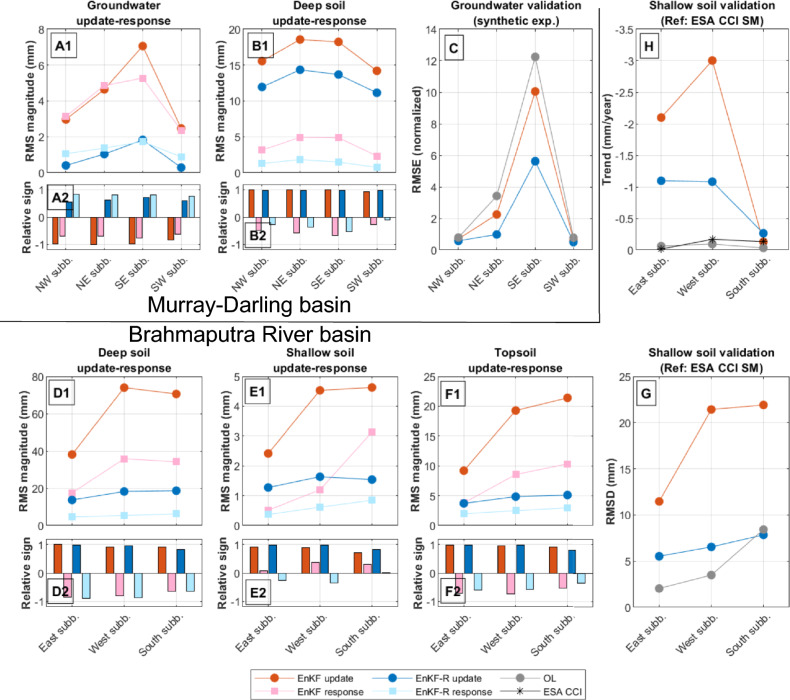
Comparison and validation plots for the Murray–Darling basin (**A**–**C**) and the Brahmaputra River basin (**D**–**H**) where (**A**) and (**B**) show the update-response dynamics of the groundwater and deep soil water, respectively; (**C**) shows the validation of the groundwater against synthetic ground truth; (**D**), (**E**) and (**F**) show the update-response dynamics of deep soil, shallow soil and topsoil water; and (**G**) and (**H**) show the validation of topsoil water against ESA CCI soil moisture data, in terms of RMSD and trend, respectively. OL (Open Loop) refers to the original model estimates. A positive/negative “relative sign” of the update means TWS and storage updates have the same/opposite sign. A positive/negative “relative sign” of the response means storage updates and responses have the same/opposite sign (see “Evaluation: measuring model update-response dynamics” section for more details). Figures generated using Matlab 2020b and modified using GIMP 3.0.2.

The update-response analysis of “Proof of concept in the Balonne River basin” section is extended to the entire Murray–Darling basin (Fig. 1A). In terms of TWS, both EnKF and EnKF-R succeed in pushing the model estimates towards GRACE observations; see Supplementary Table S3. Figure 3A1 shows the RMS of groundwater updates (dark red/blue) and responses (light red/blue) for EnKF/EnKF-R, respectively. The average relative sign of groundwater updates (relative to the sign of the TWS udpate) and the average relative sign of the response (relative to the sign of the update) are shown with the same color code in Fig. 3A2 (see “Evaluation: measuring model update-response dynamics” section for more details on the computation of these metrics). These results show that the groundwater update-response dynamics described in “Proof of concept in the Balonne River basin” section can be generalized to the entire Murray–Darling basin. The average magnitude of the updates in the whole basin is 4.3 mm for EnKF and 0.9 mm for EnKF-R; while that of the response is 3.9 mm and 1.3 mm, respectively.

The update-response dynamics of the deep soil storage compartment is found to be different from those observed for groundwater (Fig. 3B1 and B2). In this case, the EnKF updates show the same sign as the TWS updates, indicating a strong positive correlation between both variables. As a consequence, EnKF and EnKF-R lead to similar deep soil water update and response dynamics, with slightly lower magnitudes for EnKF-R (on average, $23\%$ smaller on the update and $65\%$ smaller on the response). The relative sign of the model response is less pronounced compared to groundwater for both approaches, suggesting that updates in deep soil have a minimal impact on posterior model evolution. The impacts of the DA process on shallow soil, topsoil, and surface water are shown in Supplementary Fig. S1. EnKF updates indicate that TWS is positively correlated with topsoil water and anticorrelated with shallow soil and surface water within the ensemble representation. For these compartments, the magnitude of EnKF-R updates is equal to or smaller than EnKF. The response dynamics lacks a predominant sign, which implies that the updates do not significantly influence the evolution of the model for these compartments either.

It is worth mentioning that, as by definition the EnKF-R update is localized in each sub-basin, a sub-basin localization was also applied to the classical EnKF for these experiments (see more details in “Implementation of the DA experiments” section). A non-localized EnKF experiment has also been performed, and such an approach was found to further exacerbate all the issues described so far (see results in Supplementary Fig. S2).

### Experiment 2: synthetic experiment in the Murray–Darling basin

For further comparison, we have carried out a simple synthetic experiment that allows us to validate the results against a ground truth. For this purpose, an artificial ground truth has been generated by multiplying the groundwater storage of the original model estimates by a factor of 2. We then assimilated these artificially generated TWS estimates, considering the same uncertainty as in real GRACE data, into the model. The magnitude and sign of the update-response dynamics for the synthetic experiments are provided in Supplementary Fig. S3. In general, the results are qualitatively similar to those of the real-case experiments described in “Experiment 1: Murray–Darling basin” section. Some differences can be found, probably because the synthetic TWS levels are systematically higher than the original model estimates, and therefore the update-response analysis highlights the behavior of the model under those specific conditions. For example, the relative sign of the deep soil response is notably negative for both EnKF and EnKF-R, suggesting that too much of the update is allocated to the deep soil compartment when positive TWS updates are introduced, thus corroborating the hypothesis presented at the end of “Proof of concept in the Balonne River basin” section.

The synthetic experiment allows us to validate the results against known “truth”. Our validation (Fig. 3C) shows that EnKF-R succeeds better at recovering ground truth with an average RMSD of 1.9 mm, compared to EnKF which leads to an average RMSD of 3.4 mm. As a reference, the RMSD between ground truth and the original model run is 4.3 mm, which means that EnKF reaches an accuracy that is midway between the original run and EnKF-R. The RMSD for the remaining water storage compartments is represented in Supplementary Fig. S4 and shows that the DA has a small impact on the other water storage compartments, especially when compared to the average value and variability of these storage compartments (see Supplementary Table S1).

### Experiment 3: Brahmaputra River basin

We have repeated the experiment described above for the Brahmaputra River basin (Fig. 1B) for the period 2003–2016. This basin has a very different hydrological regime that is dominated by a strong seasonality, caused by the annual monsoonal periods that affect the area. For this region, the main differences between GRACE and modeled TWS are the amplitude of the seasonality and the long-term linear trend. These differences are corrected by both EnKF and EnKF-R TWS DA experiments, thus reducing the RMSD with respect to observations; see Supplementary Table S4.

Regarding individual compartments, as shown in Fig. 1D, the deep soil, shallow soil, and topsoil compartments receive the largest updates with their update-response statistics displayed in Fig. 3D1–F1 and D2–F2. The behavior of the deep soil compartment is similar to that of the Murray–Darling; the EnKF and EnKF-R show a pronounced positive sign for the update and a counteracting response. However, the magnitude of the update and response is much smaller for EnKF-R (16.9 mm and 5.4 mm respectively) compared to EnKF (60.9 mm and 29.1 mm respectively). Very similar statistics can be observed for the topsoil water. The shallow soil shows considerably lower model responses, suggesting that the updates in this compartment might not represent any conflict with the physics of the model.

Previous studies indicate that TWS DA in this region can degrade soil moisture estimates by excessively updating the topsoil compartment^18,33^ and introducing excessive long-term trends^33^. Therefore, the topsoil estimates were validated against the ESA CCI Soil Moisture product (Fig. 3G, H). EnKF significantly degrades these estimates, increasing the RMSD from 2–8 mm (OL) to 11–22 mm (Fig. 3G). In contrast, EnKF-R maintains the estimates closer to the original, with a more limited impact (RMSD of 5–8 mm). Similar results are observed in terms of correlation coefficients (see Supplementary Fig. S5). Topsoil linear trends were also analyzed by performing a linear regression (Fig. 3H). EnKF introduces negative trends of minus 2–3 mm/year in the East and West sub-basins, overestimating the ESA CCI product’s trends (minus 0–0.2 mm/y). That of EnKF-R was found to be smaller (minus 1.1 mm/year). Therefore, EnKF-R considerably reduces the degradations that EnKF introduces in the topsoil.

Update-response dynamics for other compartments are represented in Supplementary Fig. S6. For groundwater, the effects seen in the Murray–Darling basin are repeated with less intensity, especially when considering the higher groundwater variability within the Brahmaputra River basin (Supplementary Table S2). Since the TWS-groundwater anticorrelation is weaker in this basin, less pronounced updates and responses are seen in EnKF DA compared to the Australian basin. Surface water updates show similar results to those of the Murray–Darling basin, with slightly stronger responses ($23\%$ higher on average) when using EnKF-R.

## Discussion

Previous studies show that TWS DA can improve the trend and seasonality of water storage simulations of models^15,17,20,21,24,25^. However, this improvement is sometimes achieved at the cost of degradations in individual water storage estimates such as, for example, soil moisture^25,34^, or water flux estimates such as river discharge^32^ and evapotranspiration^33^. These effects are caused by limitations of the ensemble-based TWS update disaggregation of classical DA techniques, and are not favorable for various hydrological management and monitoring purposes. Thus, the objective of this study was twofold: (i) to develop a method to further assess how TWS DA impacts individual water storage estimates; and (ii) to propose and test a novel TWS disaggregation approach that avoids the ensemble-based disaggregation of classical approaches.

Regarding the first objective, we examined how the TWS DA experiments affect individual water storage compartments (“update”) and how these water storage compartments react to the update introduced by the DA process (“response”). The sign and magnitude of the update are found to reflect the effective impact of the disaggregation process in individual water storage compartments. In contrast, the sign and magnitude of the response reveal patterns in the model reaction to the update, which can be used as an indicator of how coherent the update is with the physics represented within the model. The numerical results indicate that these metrics can identify potential issues without relying on independent measurements of individual water storage compartments and can be of particular interest for basins where such validation data are not (openly) available. Beyond the particular case of TWS DA, this approach can also be useful in analyzing the results of any DA experiment that involves the distribution of the DA update among more than one model variables.

To achieve the second objective, we proposed a new disaggregation approach that distributes the TWS updates among different model grid cells and water storage compartments in proportion to the amount of water contained in each of them (EnKF-R). By comparing EnKF-R to EnKF, both were found to lead to similar TWS estimates. However, they differ considerably in how they impact individual water storage estimates. In both the Murray–Darling and the Brahmaputra River basins, EnKF was found to introduce strong updates to various storage compartments that were likely incoherent with the physics represented within the model, as could be inferred from the strong response of the model triggered the day after the DA update. The EnKF-R, on the contrary, introduced smaller updates that led to a lighter response of the model. Validation against synthetic and real independent data showed that the strong updates of EnKF led to significant degradations for groundwater estimates (Murray–Darling) and topsoil water (Brahmaputra), while EnKF-R could reduce or avoid these degradations.

Various reasons could be behind the deficient performance of the ensemble-based disaggregation of EnKF for TWS DA, including but not limited to spurious correlations and limitations of the ensemble perturbation approach. However, it is not an objective of this work to identify why these anomalous updates occur. Various TWS DA works in the literature using a wide variety of models and EnKF variants have reported inaccurate updates of particular compartments^18,25,32,33^ and a combination of various factors might be behind the problems observed in each of these experiments. Adjustment of DA parameters, such as improving the ensemble perturbation approach and localizing or regularizing the ensemble covariance matrix, can help to improve ensemble disaggregation and achieve better results^18,35^. However, these approaches also present their own drawbacks: Setting up these strategies is a complex task, and an inappropriate setting can quickly lead to additional problems^18,37,38^. Even when set correctly, they can increase the computational cost, and they might not completely mitigate the original issue. Therefore, the proposed EnKF-R is a simpler solution to this problem.

EnKF-R not only addresses challenges related to TWS disaggregation, it also reduces the computational cost of the TWS DA. For example, the rescaling approach avoids the need to compute the covariance structure between thousands of model variables and the observed variables (matrix $C(X^-,Y^-)$ in Eq. 14, typically of dimensions $\gtrsim 10000$ at least). Instead, the disaggregation step only requires a simple rescaling of the model water storage estimates (that is, $N_b \times N_e$ multiplications between a vector and a scalar factor, where $N_b \sim 4$ and $N_e \sim 30$ are the number of sub-basins and ensemble size, respectively; see details in “Novel rescaling disaggregation scheme” section). The fact that the computation of the ensemble covariance matrix is eliminated implies that the ensemble statistics are only needed to represent the error structure of the model TWS predictions (matrix *C*(*Y*) in Eq. 8). In the case of regional experiments such as the ones considered here, this matrix has dimensions $N_b \times N_b \sim 4 \times 4$. Therefore, a smaller ensemble size of 10 or 5 members (instead of 30) might be enough to represent the variance and covariance structure of these few variables, although experiments need to be carried out to confirm this. Reducing the ensemble implies reducing the number of independent model runs and therefore notably reduces the computational cost of the DA process. It is worth mentioning that the rescaling approach can be similarly applied for both sub-basin scale and grid scale TWS DA, see more details in “Novel rescaling disaggregation scheme” section.

When implementing EnKF-R, there are still a few concerns to consider. First, the rescaling approach is implemented by treating each sub-basin independently, and can therefore lead to spatial discontinuities in the boundaries between different sub-basins, in both TWS and individual water storage estimates. This does not happen within EnKF, where each observation impacts the entire study area through ensemble correlations. The calculation of more localized rescaling factors computed in a smaller region of influence could reduce these discontinuities, but also poses the risk of introducing the smoothness of GRACE TWS observations (resolution of $\sim 300$ km) into the model. The extent of these discontinuities and possible smoothing strategies should be investigated in more detail. Second, the rescaling approach updates the TWS ensemble spread in a similar manner to EnKF by shrinking the ensemble spread and thus reducing its uncertainty. However, this is not the case for individual water storage estimates. In the present implementation, compartments that are inversely correlated with TWS can have their ensemble spread increased during the DA update (such as groundwater in the Murray–Darling basin, Supplementary Fig. S7). As a consequence, the ensemble spread of the EnKF-R results cannot be used as a meaningful indicator of the uncertainty of individual water storage estimates. Variants of the EnKF-R that include a realistic update of the ensemble spread of individual water storage compartments will be investigated in the future. Finally, the transferability of the approach to other basins remains an open question. In particular, future work will include the assessment of the EnKF-R in basins where different water storage compartments (such as snow or surface water) are more dominant.

## Method, model and data

### Data assimilation

#### Sequential ensemble-based Data Assimilation

Data Assimilation (DA) aims to update model states to push them towards the observations, all by accounting for the uncertainty of both the models and the observations. While uncertainties are typically available for observational datasets, the computation of model uncertainties is not straightforward, especially in the case of non-linear models such as land hydrological models. The ensemble approach (or Monte Carlo approach) allows one to estimate the uncertainty of (non-linear) model variables based on an ensemble of $N_e$ model realizations. The ensemble is generated by perturbing the inputs of the model, such as the forcing fields (*u*) and the parameters (*p*) according to their own uncertainties. The ensemble distribution of each model variable is then used to empirically estimate its probability distribution.

Sequential ensemble-based DA consists of a two-step process. The first step is the forward propagation of the ensemble of model states. For each member of the ensemble $(i=1,2,\ldots ,N_e)$, the water balance model $(f(\cdot ))$ is used together with the perturbed forcing fields $(u_k^{(i)})$ and parameters $(p^{(i)})$ to propagate the state of the model $x_{k}^{(i)}$ from time $k-n$ forward in time (to time $k$),

1 $$\begin{aligned} x_{k}^{(i)} = f(x_{k-1}^{(i)},u_{k-1}^{(i)},p^{(i)}). \end{aligned}$$

For example, on a monthly DA scheme using a daily model, the model propagates $n\sim 30$ days forward.

The second step, consisting of the DA update, is performed when an observation becomes available. In this step, the ensemble of model states prior to the update $(x_k^{(i)-})$ must be updated according to the information provided by an observation $(y^o_k)$ and its uncertainty $(\Sigma _{y^o})$. This will generate an updated ensemble of model states $(x_k^{(i)+})$,

2 $$\begin{aligned} {[}x_{k}^{(1)+}, x_{k}^{(2)+}, \ldots , x_{k}^{(N_e)+}] = g([x_{k}^{(1)-}, x_{k}^{(2)-}, \ldots , x_{k}^{(N_e)-}],y^o_k,\Sigma _{y^o}). \end{aligned}$$

The updated ensemble of states will then be used to initialize the next model run, which will continue until the next observation becomes available. These two steps are repeated for each new observation.

#### Ensemble Kalman filtering

The family of Ensemble Kalman Filter (EnKF)^23^ is an adaptation of the traditional Kalman filtering approach^41^ for a non-linear system, where model ensembles are used to compute model uncertainties. One of the main advantages of EnKF is that its DA update can be computed through matrix multiplication, thus simplifying its implementation and allowing for a faster computation. The model state before DA can be represented by the matrix $X_k^-$ which contains, in its $i^{\hbox {th}}$ column, the model state of the $i^{\hbox {th}}$ ensemble member, $x_k^{(i)}$. The ensemble variance-covariance matrix of the model state can be computed as

3 $$\begin{aligned} C(X_k) = \frac{1}{N_e-1} \Delta X_k \Delta X_k ^\top , \end{aligned}$$

where the matrix $\Delta X_k$ contains, in its $i^{\hbox {th}}$ column, the deviation of the ensemble member *i* from the ensemble-average of the model state, $x_k^{(i)} - \frac{1}{N_e} \sum _{i=1}^{N_e} x_k^{(i)}$.

In the classical EnKF, observations are perturbed according to their uncertainty, in order to guarantee a realistic ensemble spread in the updated model states^42^. The uncertainty of the observations is represented by its variance-covariance matrix, $\Sigma _{y^o_k}$. The observation matrix $Y_k^o$ contains each $i^{\hbox {th}}$ perturbed observation in its $i^{\hbox {th}}$ column.

Based on these matrix definitions, the updated model state $(X^+_k)$ can be computed as a matrix multiplication,

4 $$\begin{aligned} X_k^+ = X_k^- + K_k(Y^o_k - AX_k^-), \end{aligned}$$

where $K_k$ is the Kalman gain,

5 $$\begin{aligned} K_k = C(X^-_k)A^\top (AC(X^-_k)A^\top + \Sigma _{y^o,k})^{-1}. \end{aligned}$$

The matrix *A* refers to the observation operator that relates the individual model estimates (that is, individual model grid cells and water storage components) to the observed variable (here spatially averaged TWS).

#### A two-step decomposition of the EnKF

In what follows, we present a framework that separates the DA update process into two steps, allowing a better understanding of classical ensemble-based DA, and linking the concept to the EnKF-R approach in the next section (“Novel rescaling disaggregation scheme” section). The following framework is inspired by the study of^43^, but departs from the EnKF matrix equation instead of defining the problem in the joint state-observation space. This matrix-based development might be easier to follow for readers who are familiar with the equations of EnKF, and makes its computational implementation more straightforward.

For simplicity, the following developments are written for a generic timestep, and the subscript *k* has been removed. Additionally, we introduce the term “prognosis” to refer to the estimate of the observed variable (here TWS) derived from a model state. The prognosis before DA is expressed as $Y^- = A X^-$ and represents the prediction of the model of the observed variable. The prognosis after DA is calculated as $Y^+ = A X^+$ and represents the value of the observed variable in the updated state of the model.

*Step 1: update of the observation prognosis* The first step consists of updating the model prognosis solely on the basis of observations $(Y^o)$ and the prior model prognosis $(Y^-)$, as well as their respective uncertainties, without the need for any other information on individual model state variables $(X^-)$. Given the definition of the prior prognosis, $Y^- = A X^-$, the uncertainty of the prior prognosis can be computed as

6 $$\begin{aligned} C(Y^-) = A C(X^-)A^{\top }. \end{aligned}$$

Using these definitions, and replacing the Kalman gain (Eq. 5) into Eq. 4, one gets

7 $$\begin{aligned} X^+ = X^- + C(X^-)A^\top (C(Y^-) + \Sigma _{y^o})^{-1}(Y^o - Y^-). \end{aligned}$$

We can now multiply Eq. 7 by the design matrix *A* on the left side, to obtain the value of the posterior prognosis $(Y^+ = AX^+)$,

8 $$\begin{aligned} \boxed {Y^+ = Y^- + C(Y^-) (C(Y^-) + \Sigma _{y^o})^{-1}(Y^o - Y^-).} \end{aligned}$$

Thi﻿s equation reflects how model prognosis and observations are combined and weighted according to their uncertainty, following a least-squares approach (i.e. minimization of variance of the posterior prognosis) to compute the updated prognosis. The equation suggests that the posterior prognosis can be computed solely based on the prior prognosis $(Y^-)$ and the observations $Y^o$ together with their uncertainty information $(\Sigma _{y^o},C(Y^-))$. In the particular case of TWS DA, this implies that the TWS update can be computed only using TWS-related information. The distribution of the TWS among the different water storage compartments and individual grid cells $(X^-)$ does not influence the update of the TWS variable.

*Step 2: update of the individual state variables of the model* In the second step, the computed update must be distributed among the individual state variables of the model. In other words, the posterior model state $X^+$ needs to be determined. We want to show that the update of the model state can be computed solely based on the prior and posterior prognosis, as well as prior model ensemble statistics, and requires no additional information of the observations and their uncertainty.

Let us take Eq. 8 and manipulate it so as to leave all the observation-related terms $(Y^o,\Sigma _{y^o})$ on the left-hand side;

9 $$\begin{aligned} (C(Y^-) + \Sigma _{y^o})^{-1}(Y^o - Y^-) = C(Y^-)^{-1}( Y^+ - Y^-). \end{aligned}$$

The left-hand side of this equation is identical to the right-hand side of the second term in Eq. 7. Therefore, substituting this term into the Eq. 7, we get

10 $$\begin{aligned} X^+ = X^- + C(X^-)A^\top C(Y^-)^{-1}(Y^+ - Y^-). \end{aligned}$$

This equation shows that the update of the individual model variables depends only on the model prognosis before and after DA, as well as the prior model uncertainty information $(C(X^-),C(Y^-))$, and does not depend on additional information on the actual observations or their uncertainty. The equation can be further developed to facilitate its interpretation. As noted in Eq. 3, $C(X^-)$ is simply the empirical variance covariance matrix computed on the ensemble of prior model states. Recalling that $Y^- = AX^-$ (and hence $\Delta Y^- = A\Delta X^-$), the term $C(X^-)A^\top$ can be rewritten as

11 $$\begin{aligned} C(X^-)A^\top&= \frac{1}{N_e-1}\Delta X^- \Delta X^{- \top } A^\top \end{aligned}$$

12 $$\begin{aligned}&= \frac{1}{N_e-1}\Delta X^- \Delta Y^{-\top }\end{aligned}$$

13 $$\begin{aligned}&= C(X^-,Y^-). \end{aligned}$$

Here, $C(X^-,Y^-)$ represents the ensemble covariance between the individual state variables of the model and the prognosis of the model before DA. We substitute this into Eq. 10 to obtain

14 $$\begin{aligned} \boxed {X^+ = X^- + C(X^-,Y^-)C(Y^-)^{-1}(Y^+ - Y^-)}. \end{aligned}$$

This equation suggests that each of the model variables in $X^-$ will receive an update that is proportional to the ensemble covariance of that variable with the observation prognosis $(C(X^-,Y^-))$. In other words, model variables that have an ensemble distribution that is larger and more similar to that of the observation prognosis will receive a larger update.

In the particular context of TWS, this means that grid cells and water storage compartments that have a wider spread of the ensemble (that is, greater uncertainty) and a distribution of the ensemble similar to the prognosis of TWS will receive a large update with the same sign as the TWS update $(Y^+ - Y^-)$. In contrast, model variables with an ensemble distribution that is negatively correlated with the prognosis distribution of TWS will receive an update with an opposite sign. Variables with a low ensemble spread (that means low uncertainty) or that are poorly correlated with the prognosis of TWS will get a smaller update.

It is worth mentioning that variants of the EnKF such as the Ensemble Adjustment Kalman Filter^44^ or other non-Gaussian filters such as the Rank Histogram Filter or the Quantile-Conserving filter^45,46^ only differ from the EnKF in the first step and apply a similar scheme in the second step.

#### Novel rescaling disaggregation scheme

In this study, we propose an alternative approach for disaggregation in Step 2, where the distribution of the increment is performed in proportion to the amount of water contained in each of the model variables, thus avoiding the use of ensemble statistics. This approach is based on the intuitive idea that compartments that have a higher amount of water storage might also be the ones contributing the most to TWS dynamics, and, therefore, they should receive a larger portion of the DA update.

This distribution process is carried out for each ensemble member and sub-basin independently. For each ensemble member *i* and sub-basin *S*, the update of the model variables *j* belonging to that sub-basin $(j \in S)$ is performed as follows:

15 $$\begin{aligned} X(i,j)^+ - X(i,j)^- = \frac{X(i,j)^-}{Y(i,S)^-} (Y(i,S)^+ - Y(i,S)^-). \end{aligned}$$

With some simple matrix manipulations, the equation can be rewritten as $X^+(i,j) = \frac{Y(i,S)^+}{Y(i,S)^-} X^-(i,j)$. Let us now define $r(i,S) = \frac{Y(i,S)^+}{Y(i,S)^-}$, which is just the scaling factor between the posterior and prior prognosis defined for each ensemble member and sub-basin. The update of the model state can therefore be rewritten as

16 $$\begin{aligned} X^+(i,j) = r(i,S) \cdot X^-(i,j), \end{aligned}$$

which is nothing more than a rescaling of the prior model state of each ensemble member. As the rescaling factor is the same for all variables *j* belonging to each sub-basin, the whole vector containing the variables for a sub-basin can be rescaled in one unique operation,

17 $$\begin{aligned} X^+(i,j \in S) = r(i,S) \cdot X^-(i,j \in S). \end{aligned}$$

It should be mentioned that this approach is very different from an offline rescaling (i.e., a rescaling of the model state time series issued from a model run without DA). An offline rescaling does not account for the model constraints. In our approach, the rescaling is performed sequentially at the end of each month. This implies that (1) the model can integrate the update and evolve towards a natural water distribution that respects the model constraints thanks to the posterior 1-month model run, and (2) each update helps to correct the subsequent model predictions, in such a way that only small differential changes are expected to take place in each new DA update step.

It is worth mentioning that this approach can be similarly implemented in a grid scale DA framework, by substituting the sub-basins (*S*) with grid cells (*G*) in the previous formulation. In fact, this approach could be appropriate for experiments that involve assimilating any observation that has a linear relationship with the model variables (e.g., TWS represents a summation of vertical water storage compartments). Its implementation is especially adequate when the observation constitutes a spatial average of model variables, as all variables included in the area will be updated. When assimilating point observations (that is, observations corresponding to a single model grid cell), the rescaling DA should be extended to update the observation point and its surroundings within a pre-defined radius.

Finally, the rescaling approach can be combined with any other filter that can be recasted in the two-step framework presented in “A two-step decomposition of the EnKF” section.

### Model and data

The World Wide Water Resources Assessment model (W3RA)^47,48^ is a biophysical water balance model that simulates the balances of water, radiation and energy between vegetation, soil and groundwater systems. In this study, the model runs on daily steps on a grid of $0.1^{\circ } \times 0.1^{\circ }$, that is, approximately 10 km $\times$ 10 km in the Brahmaputra River basin and 8 km $\times$ 8 km in the Murray–Darling basin. A grid-based river routing model with a resolution of $0.5^{\circ } \times 0.5^{\circ }$ is used to calculate river water storage.

Princeton climate fields are used to initialize the model (https://ldas.gsfc.nasa.gov/gldas/forcing-data) and ERA5 forcing data is used to feed the model during the spin-up phase and the DA experiments (see more details in 5.3). More specifically, daily precipitation and downward surface solar radiation are derived from the ERA5-Land hourly data set^49^ and the minimum and maximum temperature are extracted from the ERA5 hourly data set at a single level^50^. All ERA5 fields have been interpolated from a native resolution of $0.25^{\circ } \times 0.25^{\circ }$ to the resolution of the model.

The monthly GRACE TWS data are derived from the GRACE level 2 (L2) products of ITSG-2018 calculated at the Institute of Geodesy of the Graz University of Technology (https://www.tugraz.at/institute/ifg/downloads/gravity-field-models/itsg-grace2018#c194122)^51,52^. During the post-processing of GRACE data, recommended corrections have been applied, which include the corrections of low-degree and order coefficients, a filtering to reduce correlated errors, and the correction of spatial leakage after the sub-basin averaging process. More details on post-processing are available in^18^.

Surface Soil Moisture product v08.1 (combined) data set^53–55^, provided by the European Space Agency Climate Change Initiative (ESA CCI, https://climate.esa.int/en/projects/soil-moisture/), is used for an independent validation of topsoil water storage estimates. The combined SSM product is derived from the combination of active scatterometers and passive radiometers and includes a temporal break correction. As the depth of the remotely detected SSM is variable^56^ and could not correspond to the definition of the topsoil within the model, the ESA CCI product was rescaled to match the mean and standard deviation of the topsoil water of the model before validation.

### Implementation of the DA experiments

This section provides practical aspects of the experimental implementation of TWS DA. For all DA experiments, an initialization is performed using Princeton climate fields over the period 1980-1999. The model is then warmed up through a spin-up phase for the period 1999-2002 with ERA5 forcing data (see more details in 5.2). As input for spin-up, precipitation is perturbed following^57^, using a Gaussian multiplicative error of $30\%$, to form an ensemble of $n_e=30$ members. Furthermore, nine model parameters are perturbed with a Gaussian multiplicative error of $40\%$ to represent the model uncertainties (see a list of the perturbed parameters and their mean value in Supplementary Table S5). As a result, we get an ensemble of $n_e=30$ perturbed water states, which are used to initialize the Open Loop (OL) run, i.e., the model run without DA. This ensemble size is in the range of those typically used in the field of TWS DA (20 – 32 ensemble members^15,17,20,25^).

Before each DA experiment, the long-term average of the OL TWS estimates is added to the zero-mean GRACE TWS anomalies, to remove the offset between GRACE (mass anomalies) and the absolute TWS values of the model^17,58^. For all DA experiments, the monthly TWS fields were assimilated as spatially averaged fields over the sub-basins represented in Fig. 1. The size of these sub-basins was chosen to match the approximate resolution of monthly GRACE TWS observations ($\sim 300$ km). The assimilation on the sub-basin scale was chosen over the grid scale because the treatment of the signal and noise of TWS, such as accounting for filtering, scaling, and spatial leakage, could be done more rigorously on the sub-basins^59^. However, all the approaches described in this study can also be applied in a grid scale DA framework (see more details in “Novel rescaling disaggregation scheme” section).

Before each DA update, the equivalent snow water content and the vegetation water content were deducted from the observed TWS value, and these compartments were also excluded from the calculation of the TWS prognosis and the model update process. This was done to simplify the model output and the posterior analyses. Monthly TWS observations are assimilated at the end of each month as a monthly averaged value. This means that before performing the DA step an average of all the model states of the previous month is computed, and the DA is later applied over this temporally averaged state. The results represented in Fig. 2A, B, D, and E, as well as all of the statistics presented in this manuscript refer to the temporally averaged updated model states. After each DA step, and before starting with the next model run, the monthly update is applied on the last day of the month, to correct subsequent model predictions (see a visual representation of the updates in Fig. 2C). In some cases, especially when the TWS levels are low, the DA update can result in negative water storage values for the ensemble members that have the lowest storage values. This issue is addressed by setting all negative water storage values to zero after the application of the DA update. This approach is similar to that used in previous studies^15,28^.

In this study, covariance between the sub-basin averaged GRACE TWS observations is considered to be zero to treat each sub-basin independently. Finally, because the EnKF-R applies the DA update to each sub-basin independently, and to guarantee a fair comparison, sub-basin localization is also applied to the EnKF. This means that all covariances between model variables belonging to different sub-basins are set to 0. As a consequence, the prognosis uncertainty $C(Y^-)$ is also a diagonal matrix. However, a non-localized EnKF experiment is also performed for comparison in the Murray–Darling basin experiment, as mentioned in “Experiment 1: Murray–Darling basin” section. The results of the non-localized EnKF experiment are provided in the Supplementary Fig. S2.

### Evaluation: measuring model update-response dynamics

In this study, we also propose a novel evaluation approach that consists of measuring the model update-response dynamics. To our knowledge, no other study in the field of TWS DA has used this approach before. The DA update of the model state in the timestep $t_k$ is defined as the difference between the model state after and before the DA update,

18 $$\begin{aligned} U_k = X^+_k - X^-_k. \end{aligned}$$

The model response to the update of the timestep $t_k$ is measured as the difference between the value of that variable for the day following the DA update $(t_{k+1})$ and the updated value in time $t_k$,

19 $$\begin{aligned} R_k = X_{k+1}^- - X^+_k. \end{aligned}$$

In other words, the update term reflects how the DA update modifies the value of that particular variable, and the model response reflects the model reaction to the update in the first timestep after the DA update. If a DA update is consistent with the constraints of the model and its natural behavior, the response of the model should be smooth and follow the dynamics of the model. However, if a DA update makes the model deviate too much from its natural dynamics (for example, if too much water is added to shallow soil and at the same time water is removed from the deep soil), the model might present a strong response that counteracts the model update and evolves towards a more balanced model states (in this case, very fast percolation from shallow soil to deep soil).

The update and response direction and intensity have been quantified by computing the Root Mean Square (RMS) magnitude of the time series as well as their relative sign. The root mean square magnitude of updates in a storage compartment *S* is calculated as $RMSM(U_S) = \sqrt{\frac{1}{T}(\Sigma_{k\in K_{DA}}{U_{k,S}^2})}$, where $K_{DA}$ contains all the timesteps *k* where the monthly DA update is applied. A similar calculation is applied for the RMS magnitude of the response. The relative sign of the update is calculated as the average of the multiplication of the sign of the TWS update and the sign of the storage update, $\frac{1}{T}\Sigma _{k\in K_{DA}}{\hbox {sign(}U_{k,TWS}\hbox {)}\cdot \hbox {sign(}U_{k,S}\hbox {)}}$, where *T* is the amount of DA updates. The relative sign of the response is calculated as the average of the multiplication of the sign of the storage update and the sign of the storage response $\frac{1}{T}\Sigma _{k\in K_{DA}}{\hbox {sign(}U_{k,S}\hbox {)}\cdot \hbox {sign(}R_{k,S}\hbox {)}}$.

Additional validations are performed against independent data by computing Root Mean Square Difference (RMSD) and Pearson’s correlation coefficient statistics, as well as long-term trends computed using linear regression. Before the computation of these statistics, model estimates are averaged over all the ensemble members and over each sub-basin.

## Supplementary Information

Supplementary Information.

## Data Availability

The results of the experiments included in this study are available in a Zenodo repository with doi 10.5281/zenodo.15632536 (https://zenodo.org/records/15632536).
