# Supplementary material for: An ensemble Kalman filter with rescaling disaggregation for assimilating terrestrial water storage into hydrological models
Source: Sci Rep. 2025 Aug 6;15:28675. doi: 10.1038/s41598-025-13602-2 (PMC12325959; doi:10.1038/s41598-025-13602-2)
Supplement: Supplementary file 1 — Supplementary Information. [file 41598_2025_13602_MOESM1_ESM.pdf]

An Ensemble Kalman Filter with Rescaling Disaggregation for Assimilating Terrestrial Water Storage into Hydrological Models
-
Supplementary Information

Leire Retegui-Schiettekatte[a,c], Maike Schumacher[a], Fan Yang[a], Henrik Madsen[b], Ehsan Forootan[a]

[a] *Geodesy Group, Department of Sustainability and Planning, Aalborg University, Rendsburggade 14, 9000, Aalborg, Denmark,*
[b] *DHI A/S, Agern Allé 5, 2970 Hørsholm, Denmark,*
[c] *Corresponding author leirears@plan.aau.dk*

S1 Sign of the individual water storage update under the EnKF-R approach.

Following the concept behind the rescaling approach, the sign of the update of individual components could be expected to be the same as the sign of the TWS. However, the relative sign of the update reveals that this is not always the case.

This effect can be explained by the fact that these update dynamics have been computed over ensemble-averaged dynamics. For each ensemble, the updates of the model variables will have the same sign as the TWS update. However, the effective update of the ensemble-averaged TWS estimate might not have the same sign as that of the effective update of the ensemble-averaged individual compartments.

Table S1: Mean and standard deviation of individual water storage compartment time series in the Murray-Darling basin.

	Mean (mm)			
	NW subb.	NE subb.	SE subb.	SE subb.
Groundwater	0.4	3.0	10.9	0.6
Deep water	142.6	164.9	170.2	133.5
Shallow water	3.1	5.9	8.9	4.2
Topsoil water	28.4	35.8	39.6	33.2
Surface water	0.1	0.1	0.3	0.0
	STD (mm)			
	NW subb.	NE subb.	SE subb.	SE subb.
Groundwater	0.7	2.2	9.7	0.8
Deep water	15.0	15.5	17.1	12.9
Shallow water	4.3	5.6	7.4	4.7
Topsoil water	14.2	12.7	14.3	16.0
Surface water	0.3	0.3	0.6	0.2

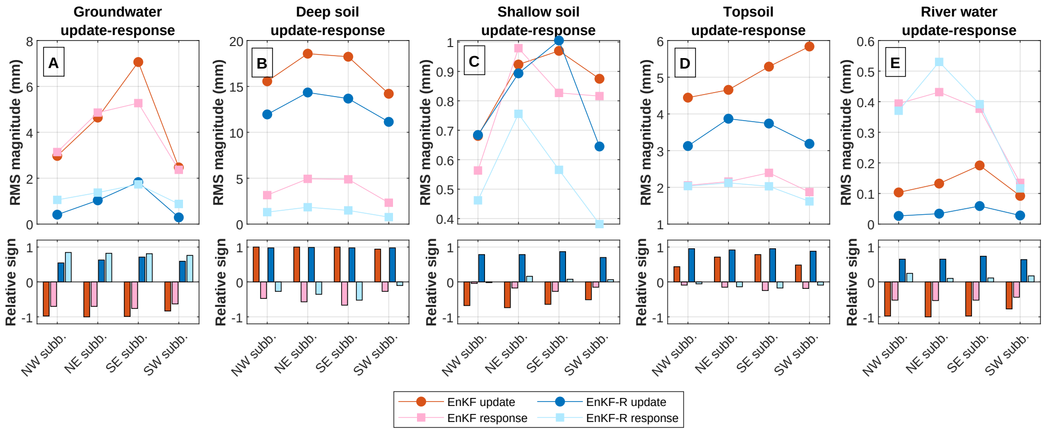

Figure S1: Update-response dynamics in the **Murray-Darling basin** during the **real-case** TWS DA experiment. A) Groundwater compartment; B) deep water compartment; C) shallow water compartment; D) topsoil water compartment; and D) surface water compartment.

Table S2: Mean and standard deviation of individual water storage compartment time series in the Brahmaputra River basin.

	Mean (mm)		
	East subb.	West subb.	South subb.
Groundwater	220.9	305.8	272.8
Deep water	183.2	188.6	191.3
Shallow water	16.1	15.8	13.7
Topsoil water	47.4	49.0	50.7
Surface water	57.0	125.8	119.2
	STD (mm)		
	East subb.	West subb.	South subb.
Groundwater	114.6	180.9	168.1
Deep water	13.4	18.0	22.3
Shallow water	3.6	6.1	10.5
Topsoil water	8.0	11.4	17.7
Surface water	36.8	84.1	78.1

Table S3: RMSD of OL TWS, EnKF TWS and EnKF-R TWS with respect to the monthly GRACE TWS, in the Murray-Darling basin DA experiment.

	RMSD difference with monthly GRACE TWS (mm)			
	NW subb.	NE subb.	SE subb.	SE subb.
OL TWS	35.31	41.20	46.78	39.65
EnKF TWS	10.23	10.27	9.46	11.40
EnKF-R TWS	8.78	6.99	5.33	10.47

Table S4: RMSD of OL TWS, EnKF TWS and EnKF-R TWS with respect to the monthly GRACE TWS, in the Brahmaputra River basin DA experiment.

	RMSD difference with monthly GRACE TWS (mm)		
	East subb.	West subb.	South subb.
OL TWS	106.7	152.3	85.8
EnKF TWS	31.6	40.1	34.3
EnKF-R TWS	30.9	27.8	31.5

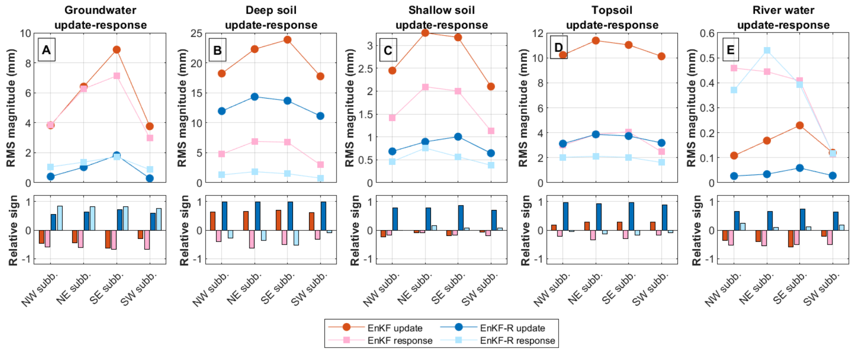

Figure S2: Same plot as S1, but considering a **non-localized** classical EnKF implementation.

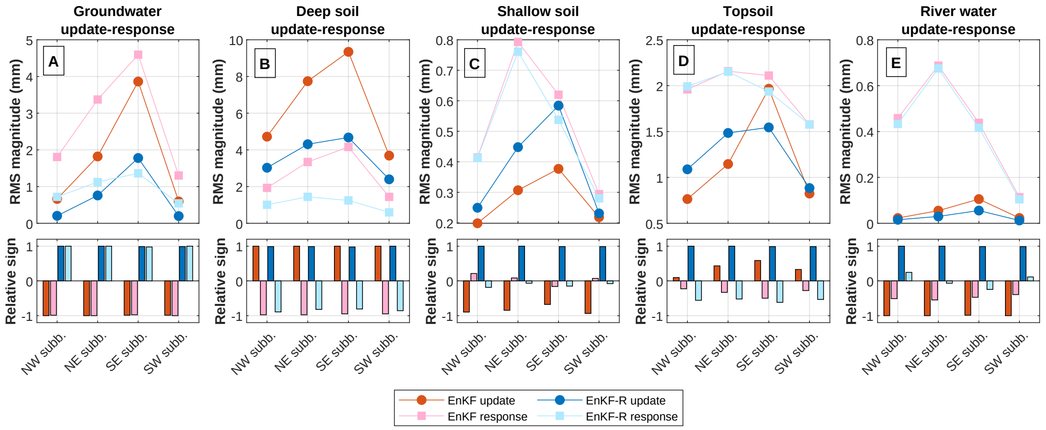

Figure S3: Update-response dynamics in the **Murray-Darling** basin during the **synthetic** TWS DA experiment. A) Groundwater compartment; B) deep water compartment; C) shallow water compartment; D) topsoil water compartment; and D) surface water compartment.

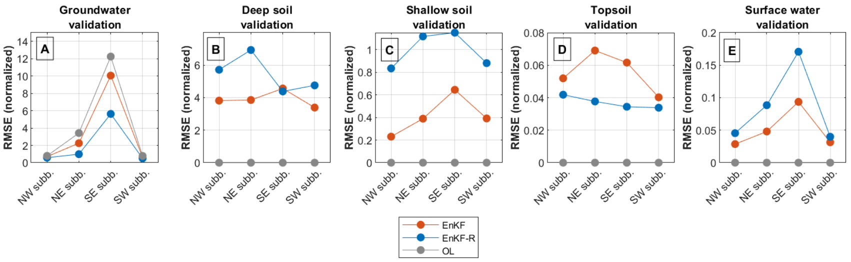

Figure S4: RMSE of individual model compartments with respect to ground truth, in the **Murray-Darling basin** during the **synthetic** TWS DA experiment. A) Groundwater compartment; B) deep water compartment; C) shallow water compartment; D) topsoil water compartment; and D) surface water compartment.

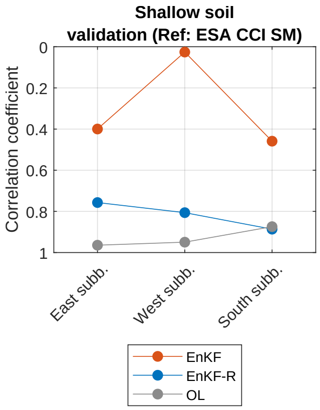

Figure S5: Correlation coefficient of shallow water estimates of OL, EnKF and EnKF-R, with respect to ESA CCI soil moisture, in the **Brahmaputra River basin**.

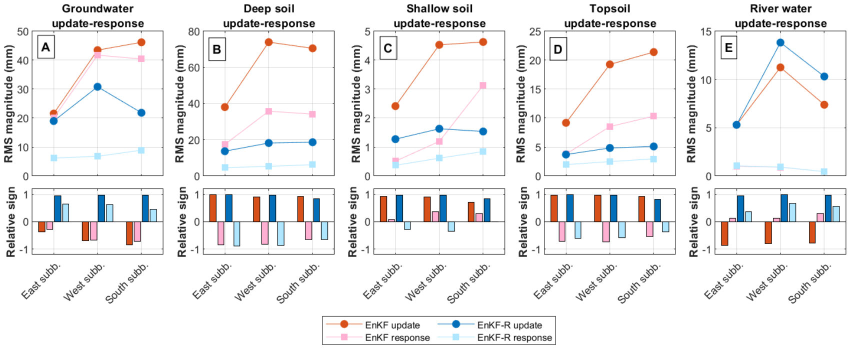

Figure S6: Update-response dynamics in the **Brahmaputra River basin** during the TWS DA experiment. A) Groundwater compartment; B) deep water compartment; C) shallow water compartment; D) topsoil water compartment; and D) surface water compartment.

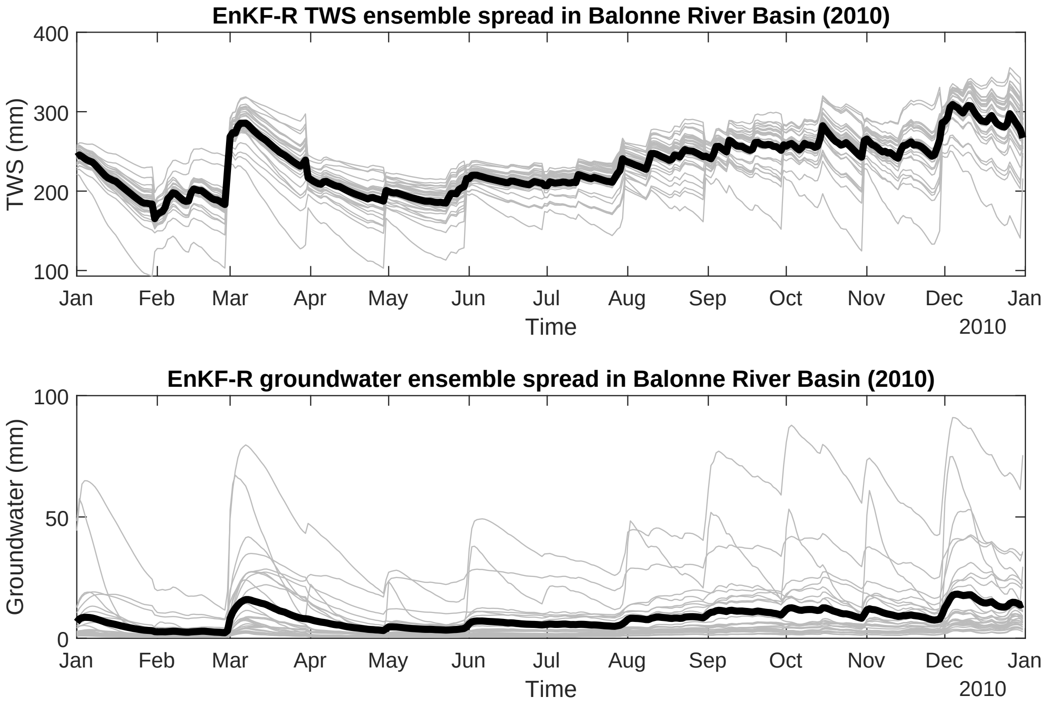

Figure S7: Ensemble spread of TWS and groundwater estimates issued from TWS DA through EnKF-R, in the Balonne River basin for year 2010. Although the ensemble spread of TWS is reduced after each DA update, due to the negative ensemble-correlation between TWS and groundwater, the ensemble spread of groundwater is increased.

Parameter	Description	Mean HRU1	Mean HRU2
β	Coefficient describing rate of hydraulic conductivity increase with water content (-)	1.21	13.55
F_{ER0}	Average ratio of wet canopy evaporation rate and rainfall rate for full canopy cover (-)	0.14	0.04
F_{sEmax}	Maximum soil evaporation fraction (-)	0.72	0.95
I_0	Initial retention capacity (mm)	25.28	7.35
P_{ref}	Reference event precipitation for runoff generation (mm d-1)	148.1	732.6
S_V	Canopy storage capacity per unit leaf area (mm)	0.04	0.13
S_{0FC}	Accessible top soil water storage at field capacity (mm)	9.20	88.38
S_{dFC}	Accessible deep soil water storage at field capacity (mm)	458.9	320.56
S_{sFC}	Accessible shallow soil water storage at field capacity (mm)	3.22	35.26

Table S5: Perturbed W3RA model parameters, description and mean value for HRU1 (tall, deep-rooted vegetation) and HRU2 (short, shallow-rooted vegetation). The ensemble perturbation approach is described in Section 5.3 of the main manuscript.
